# Extraction optimization and screening of antioxidant peptides from grass carp meat and synergistic–antagonistic effect

**DOI:** 10.1002/fsn3.2765

**Published:** 2022-03-25

**Authors:** Xiao‐yan Jia, Min‐fang Zhu, Lu Zhang, Tian‐Xin Ma, Yi‐hua Li, Wen‐sheng Sheng, Zong‐cai Tu

**Affiliations:** ^1^ 47861 National R&D Center of Freshwater Fish Processing, and Engineering Research Center of Freshwater Fish High‐value Utilization of Jiangxi College of Life Science Jiangxi Normal University Nanchang China; ^2^ Jiangxi Deshang Pharmaceutical Research Institute Co., Ltd. Yichun China; ^3^ 47861 State Key Laboratory of Food Science and Technology Nanchang University Nanchang China

**Keywords:** antioxidant peptides, grass carp, identification, synergistic effect, two‐step hydrolysis

## Abstract

Grass carp (*Ctenopharyngodon idellus*) is one of the three most cultivated freshwater fish around the world, but it is mainly consumed afresh, so only a small part of them are processed into salted fish or snack food. This research was performed to prepare and screen antioxidant peptides from grass carp muscle to promote its high‐value utilization. The parameters of double‐enzyme two‐step hydrolysis were optimized, the peptides with the highest ABTS.^+^ scavenging ability were enriched and identified by Sephadex G‐25 and LC‐Q‐Orbitrap‐MS/MS. The synergistic–antagonistic effect among identified peptides was also investigated. The optimized conditions were hydrolyzed with protamex (10,000 U/g) at pH 8.0, 50°C for 3 h, followed by hydrolysis with alcalase (6,000 U/g) at pH 9.0, 50 °C for 2 h, and the protein–liquid ratio was 4%. The hydrolysates were further fractionated to obtain five fractions, in which fraction 3 (F3) exhibited the strongest ABTS.^+^ and $O_{2}^{\cdot ‐}$ scavenging ability with the IC_50_ values of 0.11 and 0.47 mg/ml, respectively. Twelve novel antioxidant peptides were identified, in which VAGW possessed the highest activity (139.77 μmol GSH/g). Significantly synergistic effects were observed on the two and three peptides’ combination among VAGW, APPAMW, LFGY, FYYGK, and LLLYK, while the C‐terminal tryptophan (Trp) played an important role in the synergism. This study found that grass carp muscle hydrolysates can be potential natural antioxidants in functional products. The synergistic effects among peptides may provide a perspective for the combined application of peptides.

## Graphical abstract

The conditions of double‐enzyme two‐step hydrolysis were optimized. Twenty antioxidant peptides were identified, of which VAGW showed the best antioxidant activity. The combination of two and three peptides showed a good synergistic effect.

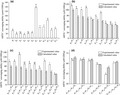

## 1 INTRODUCTION

Reactive oxygen species (ROS), for instance hydroxyl free radical, hydrogen peroxide, and singlet oxygen are normal products of cellular respiration or as a response of human body to ultraviolet (UV) radiation, chemical agents, and thermal stress. But overformation or accumulation of ROS will trigger oxidative stress, which is related to many human diseases, such as cardiovascular diseases (Petyaev et al., 2018), cancers (Diehn et al., 2009), diabetes, aging and neurodegenerative diseases (Sarmadi & Ismail, 2010), etc. Therefore, it is indispensable to obtain additional protection to balance the oxidation state (Jaouad & Torsten, 2010), at this point, antioxidants become the primary option. Although synthetic antioxidants such as butylated hydroxytoluene, butylated hydroxyanisole, propyl gallate, and tertiary butylhydroquinone have good antioxidant activities, their use in diet is limited because of toxic and side effects (Lobo et al., 2010). Natural antioxidants, especially antioxidant peptides derived from food, have attracted widespread attention, since they can be isolated from countless sources and have the advantages of low side effects and high absorption (Sarmadi & Ismail, 2010).

Antioxidant peptides are usually composed of 2–20 amino acid residues, which can be released by enzymatic hydrolysis during gastrointestinal digestion or food processing. Up to date, a large number of antioxidant peptides have been isolated and identified from aquatic protein, for example *Raja porosa* cartilage (Pan et al., 2016), *Pseudosciaena crocea* muscle (Chi et al., 2015), *Setipinna taty* (Song et al., 2015), and fish gelatin (Zamorano‐Apodaca et al., 2020). However, as the majority of reported bioactive peptides were derived from seafood proteins, researches on the antioxidant peptides from freshwater fish are much less.

Grass carp (*Ctenopharyngodon idellus*), belonging to the family *Cyprinidae,* is not only one of the seven major freshwater fish species in China (Yang et al., 2020), but it is also one of the four most cultivated freshwater fish around the world. The annual production of cultured grass carp in China exceeded 5.50 million tons in 2018 (China, 2019). According to the abundance in bioactive proteins and unsaturated fatty acids, grass carp is a traditionally high‐quality resource (Qin et al., 2020). Its muscle and skin hydrolysates were reported to show angiotensin‐I converting enzyme (ACE) inhibition (Yi et al., 2016) and antioxidant activities (Chen et al., 2016). The antilisterial peptides derived from grass carp proteins can efficiently inhibit the growth of *L. monocytogenes* in surimi noodle (Xiao & Niu, 2015). Furthermore, a novel excellent ACE inhibitory peptide Val‐Ala‐Pro (Chen et al., 2012) and a potent antioxidant peptide Pro‐Ser‐Lys‐Tyr‐Glu‐Pro‐Phe‐Val (Zhao et al., 2008) were isolated from the grass carp protein hydrolysates prepared with alcalase. However, reports regarding the screening and characterization of antioxidant peptides from grass carp muscle are much less than skin.

Enzymatic hydrolysis is the most common method for preparing bioactive peptides because of the milder and controllable process, which includes single‐, double‐, and multi‐enzyme hydrolysis, the latter two hydrolyses can be further divided into step‐by‐step and mixed hydrolysis (Sharma et al., 2020; Liu et al., 2016). Double‐enzyme hydrolysis possesses the advantages of more cleavage sites, higher hydrolysis degree, and simpler enzymatic hydrolysis process. For example, the degree of hydrolysis (DH) of *spirulina platensis* protein catalyzed by alkaline and papain was 25.47% and 21.73%, respectively. It was increased to 32.90% when the protein was hydrolyzed by alkaline and papain sequentially (Sun et al., 2016). The 2,2‐diphenyl‐1‐picrylhydrazyl (DPPH) scavenging ability of corn protein alkaline–flavourzyme two‐step hydrolysates was 2.59‐fold of that hydrolyzed by flavourzyme (Jin et al., 2016).

The purpose of this work was to optimize the double‐enzyme two‐step hydrolysis parameters of grass carp muscle using the ABTS.^+^ scavenging ability and degree of hydrolysis (DH) as indicators, and to screen the antioxidant peptides via chromatography separation and LC‐Q‐Orbitrap‐MS/MS. The identified peptides were synthesized to evaluate the antioxidant activity, and to analyze the synergistic and antagonistic effects. Finally, the relationship between chemical structure and antioxidant ability of tested peptides was analyzed. This work would provide technical and theoretical support for further utilization of grass carp proteins as potential natural antioxidants in functional products.

## 2 MATERIALS AND METHODS

### 2.1 Materials

Fresh grass carp was purchased from Rainbow mall (116°02′E, 28°67′N) in Nanchang, Jiangxi province, China. Alcalase and protamex were provided by Novozymes (Bagsvaerd, Denmark) and Ruiyang Biotechnology Co. Ltd (Jiangsu, China), respectively. Formic acid, 2,2′‐azino‐bis(3‐ethylbenzothiazoline‐6‐sulfonic acid) (ABTS), and trifluoroacetic acid (TFA) were purchased from Sigma‐Aldrich (Sigma, St. Louis, MO). Glutathiose (GSH), ferrozine, l‐glutathione reduced, pyrogallol, and other reagents were obtained from Solarbio (Beijing, China). Sephadex G‐25 was purchased from GE Healthcare (Pittsburgh, USA). Formic acid and TFA were of chromatographic grade, while other reagents were of analytic grade.

### 2.2 Optimization of hydrolysis conditions

Protamex–alcalase stepwise hydrolysis was selected as an appropriate method based on the results of our pre‐experiments, which showed the strongest ABTS.^+^ scavenging ability compared with other double‐enzyme combinations of alcalase, neutrase, flavourzyme, and protamex (shown in Figure S1).

Fresh grass carp meat collected from the back was minced, and the content of crude lipid (1.68 ± 0.55% [fresh weight]) was determined by the Soxhlet extraction method. Therefore, the minces were mixed with distilled water directly without degreasing treatment. This mixture was then hydrolyzed with protamex at an enzyme/substrate [E/S] ratio of 10,000 U enzyme/g protein, pH of 7.0, and temperature of 50°C for 3 h (Tkaczewska et al., 2020). Following, the second‐step hydrolysis was operated with alcalase. According to the ABTS.^+^ scavenging ability of hydrolysates, the parameters of alcalase hydrolysis, including initial the protein–liquid ratio (1%, 2%, 3%, 4%, 5%), alcalase–substrate ratio [E/S] (6,000, 8,000, 10,000, 12,000, 14,000 U/g), hydrolysis temperature (0, 40, 50, 60, 70 °C), hydrolysis time (1, 2, 3, 4, 5 h), and pH (6, 7, 8, 9, 10), were compared to achieve the optimal dual‐enzyme stepwise hydrolysis conditions. The blank samples (that contained all of the reagents without grass carp meat) were prepared in parallel. The protein–liquid and alcalase–substrate ratios (U/g protein) were calculated based on the protein content in minces detected by Kjeldahl's method (Marcia & Sebranek, 1993). After enzymatic hydrolysis, the solutions were boiled for 10 min to inactivate the enzyme and centrifuged at 602 *g* for 10 min, and the supernatants were gathered and used for antioxidant ability analysis.

### 2.3 Determination of the degree of hydrolysis

The degree of hydrolysis (DH) of all hydrolysates was calculated from the ratio of α‐amino nitrogen to total nitrogen. The amino nitrogen content (X_1_) was determined by the formaldehyde titration method (Lin et al., 2013). The total nitrogen content (X_2_) was measured with the Kjeldahl method (Marcia & Sebranek, 1993). The DH was calculated according to the following equation:

(1) $$\text{DH}\left(\%\right)=\frac{x_{1}}{x_{2}}\times 100\%$$

### 2.4 Amino acid composition analysis

The grass carp hydrolysates (GCHs) prepared with the optimal hydrolysis conditions were lyophilized and subjected to amino acid composition analysis according to a reported method with slight modifications (Siswoyo et al., 2011). The GCHs were hydrolyzed with 6 mol/L HCl in a hydrolysis tube under 110°C for 24 h. Then, the volume was adjusted to 25 ml with distilled water, and 1 ml of the mixed sample was dried under reduced pressure and redissolved in sodium citrate buffer solution (1.0 ml, pH 2.2). Finally, the sample was filtered through a 0.22‐μm membrane and subjected to an Automatic Amino Acid Analyzer (L‐8900, Hitachi, Japan).

### 2.5 Determination of molecular weight distribution

The molecular weight (MW) distribution of GCHs was determined using an Agilent 1260 Infinity II LC HPLC System (Agilent, Palo Alto, CA) equipped with a Waters XBridge Protein BEH 125 Å SEC column (3.5 μm, 7.8 × 300 mm). Samples were eluted with 40% acetonitrile containing 0.1% TFA for 30 min at a flow rate of 0.4 ml/min. The detected wavelength and injection volume were 220 nm and 10 μl, respectively. Cytochrome (MW: 12,384 Da), aprotinin (MW: 6511.51 Da), bacitracin (MW: 1422.69 Da), l‐glutathione oxidized (MW: 612.63 Da), and hydroxyproline (MW: 131.13 Da) were prepared to plot the linear standard curve of log MW versus retention time. All samples were passed through a 0.22‐μm membrane (Millipore, USA) before HPLC analysis.

### 2.6 Separation by gel filtration chromatography

The GCHs were dissolved in distilled water and separated on a Sephadex G‐25 gel filtration column (Ф 1.6 cm × 80 cm) (Haofeng et al., 2021). The sample solution was loaded onto the pre‐equilibrated column and eluted by ultrapure water at a flow rate of 0.4 ml/min. The elution was collected at 5‐min intervals by an automated fraction collector and detected at 220 nm. Ultimately, five fractions were collected and freeze‐dried to evaluate the ABTS.^+^ scavenging capacity, $O_{2}^{\cdot ‐}$ scavenging ability, and Fe^2+^ chelating ability.

### 2.7 Antioxidant ability analysis

The ABTS.^+^ radical scavenging assays were carried out according to the methods reported by Yang et al. (2021). Sample solutions (50 μl) at suitable concentrations were reacted with 150 μl of freshly diluted ABTS.^+^ solution in a 96‐well microplate at 25°C for 30 min. The absorbance (A_i_) at 734 nm was measured by a microplate reader (BioTek, USA). GSH was applied as positive control. The percentage inhibition was calculated using the following formula:

(2) $${\text{ABTS}}^{\cdot +}\;\text{inhibition}\;\left(\%\right)=\frac{\left(\text{Ac}‐\text{Ab})‐(\text{Ai}‐\text{Aib}\right)}{\left(\text{Ac}‐\text{Ab}\right)}\times 100$$

where Ab is the absorbance of blank group, Ac is the absorbance of control group, Ai is the absorbance of sample group, and Aib is the absorbance of sample blank group with radical replaced by distilled water. The concentration required to scavenge 50% of ABTS.^+^ (IC_50_ value) was expressed as mg/ml.

The $O_{2}^{\cdot ‐}$ scavenging ability and Fe^2+^ chelating ability of GCHs and their fractions were measured based on the methods reported by Guo et al. (2009) and Hu et al. (2012), respectively, and calculated with Equation (2). GSH was used as positive control, while the concentration required to scavenge 50% of $O_{2}^{\cdot ‐}$ or chelate 50% of Fe^2+^ (IC_50_ value) was expressed as mg/mL and used to evaluate the activity.

### 2.8 Identification of peptide sequences

The peptide fraction exhibiting the strongest ABTS.^+^ scavenging activity was used for further identification of amino acid sequence through a Nano‐LC‐Orbitrap‐MS/MS system (Ma et al., 2003). Peptides were eluted on an AcclaimR PepMap100 guided column (100 μm × 20 mm, C18, 5 μm, 100 Å) and an AcclaimR PepMap RSLC analysis column (50 μm × 150 mm, C18, 2 μm, 100 Å) at a flow rate of 220 nl/min. The mobile phase A was 0.1% formic acid in water and mobile phase B was 0.1% formic acid in acetonitrile. The gradient elution program was: 0–2 min, 4–12% B; 2–25 min, 12%–22% B; 25–32 min, 22%–32% B; 32–37 min, 32%–75% B; 37–40 min, 75% B.

The mass data were acquired on an Orbitrap Q‐Exactive mass spectrometer controlled by Xcalibur 2.2 SP1 software under positive ion mode. The mass spectrometry (MS) spectra were obtained at a resolution of 70,000 with the target value of 3e6 and scan range of m/z 250–1,350. Peptide fragmentation was performed via higher‐energy collision dissociation (HCD), while MS/MS spectra were acquired at a resolution of 17,500 and a target value of 5e4. PEAKS Studio 7.0 software combined with de novo sequencing was used to process the MS/MS data. The identified peptides meet the false discovery rate (FDR) ≤ 5% and the average local confidence score (ALC) ≥ 95%.

### 2.9 Peptide synthesis and synergistic effect analysis

In this research, 15 identified peptides were selected based on the structure–activity relationships of antioxidant peptides and synthesized by Jier Biotechnology Co. Ltd (Shanghai, China) (Li et al., 2020; Liu et al., 2016; Rajapakse et al., 2005). The purity of all synthesized peptides was over 95%. The ABTS.^+^ scavenging ability of synthesized peptides was tested according to the method described above.

The synergistic or antagonistic effect of synthesized peptides was investigated according to the method of Becker et al. (2007). The concentration of all peptides was fixed at 2 mg/ml, then one, two, three, or four peptides with different amino acid sequences were mixed in an equal volume to obtain the combined peptides. The experimental value (EV) was expressed as GSH equivalent value (μmol GSH/mg peptide). The calculated value (CV) of combined peptides was calculated based on the average GSH equivalent value of each single peptide. Higher EV than CV indicates a synergistic effect, while lower EV implies an antagonistic effect.

### 2.10 Statistical analysis

All samples were analyzed in triplicate, and the data were expressed as mean ± standard deviation (*SD*). Statistical analysis was carried out by one‐way analysis of variance (ANOVA) and Tukey's test with SPSS version 17.0, *p* < .05 was regarded as significant.

## 3 RESULTS AND DISCUSSIONS

### 3.1 Optimization of alcalase second‐step hydrolysis parameters

**FIGURE 1:**
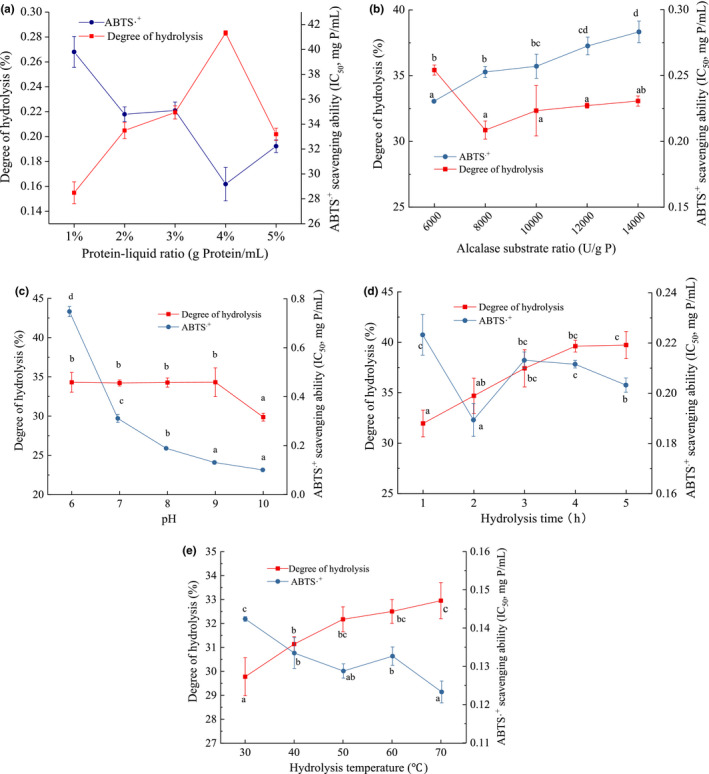
Effects of the protein–liquid ratio (a), alcalase–substrate ratio (b), hydrolysis pH (c), hydrolysis time (d), and hydrolysis temperature (e) on the ABTS.^+^ scavenging capacity and degree of hydrolysis of grass carp two‐step hydrolysates

The mincing of grass carp was hydrolyzed first by protamex, and the hydrolysates were then subjected to further hydrolysis with alcalase. The hydrolysis parameters of protamex were chosen based on the previous report (Tkaczewska et al., 2020). According to the ABTS.^+^ scavenging ability and DH, the protein–liquid ratio, alcalase addition, hydrolysis temperature, hydrolysis time, and pH for the second‐step hydrolysis of alcalase were optimized subsequently to obtain the most suitable parameters, and the results are presented in Figure 1. Higher IC_50_ value indicates lower radical scavenging ability, whereas a higher DH suggests better hydrolysis efficacy. However, simply positive or negative correlation between DH and ABTS.^+^ scavenging ability was not observed among all single‐factor experiments, except for the protein–liquid ratio. This suggests that higher DH does not indicate stronger radical scavenging ability. Therefore, ABTS.^+^ scavenging ability was considered as the evaluation index to optimize parameters for screening antioxidant peptides. The IC_50_ value decreased gradually when the protein–liquid ratio was increased from 1% to 4%, implying that a high protein–liquid ratio results in stronger ABTS.^+^ scavenging ability (Figure 1a). But a decreasing trend was observed when the alcalase–substrate ratio was increased from 6,000 to 14,000 U/g P (Figure 1b). Therefore, for the next experiments, the optimal protein–liquid ratio and alcalase–substrate ratio were 4% and 6,000 U/g P, respectively.

As for the hydrolysis pH, the ABTS.^+^ scavenging ability of hydrolysates was improved with increasing pH. The lowest IC_50_ value (0.10 mg P/mL) was detected at pH 10.0, but insignificant difference was observed between the IC_50_ values of hydrolysates prepared at pH 9.0 and 10.0 (Figure 1c). Thus, 9.0 was chosen as the suitable pH for the following experiments.

As shown in Figure 1d, the second‐step hydrolysis with alcalase for 2 h exhibited the highest ABTS.^+^ scavenging ability, while the overlong hydrolysis time reduced the scavenging ability of hydrolysates. This could be due to prolonged enzymatic hydrolysis time and cleavage of the antioxidant peptides into shorter peptide with low activity (Chen et al., 2012). The influence of hydrolysis temperature showed the same change in tendency (Figure 1e). The sample hydrolyzed at 70°C presented the lowest IC_50_ value, but it showed insignificant difference with that at 50°C (*p* > .1). This is consistent with the finding of Chen et al. (2018) that the ACE inhibition of hydrolysates was decreased when the proteolysis time and temperature increased over a certain value. Hence, the suitable hydrolysis time and temperature of alcalase were set at 2 h and 50°C, respectively.

Based on the stepwise optimization results of single‐factor experiments, the optimal second‐step hydrolysis parameters for alcalase were: protein–liquid ratio, 4%; pH, 9.0; enzyme–substrate ratio, 6,000 U/g; hydrolysis temperature, 50°C; time, 2 h. Finally, grass carp meat was first hydrolyzed with protamex at a protein–liquid ratio of 4%, enzyme/substrate ratio of 10,000 U/g, pH of 7.0, and temperature of 50°C for 3 h. Then, the pH was adjusted to 9.0, alcalase was added at an enzyme/substrate ratio of 6,000 U/g to start the second‐step hydrolysis progress at 50°C for 2 h. After centrifugation at 782 *g* for 10 min, the supernatant was gathered and freeze‐dried to obtain the grass carp hydrolysates (GCHs). The ABTS.^+^ scavenging ability, $O_{2}^{\cdot ‐}$ scavenging ability, and Fe^2+^ chelating ability of GCHs were evaluated. An obvious dose‐dependent relationship was observed in the three antioxidant models (data were not shown), and the calculated IC_50_ values are shown in Table 1. The IC_50_ value (0.21 mg/ml) for ABTS.^+^ scavenging ability was much lower than that without optimization (0.31 mg/ml, Figure S1), suggesting a good optimization efficacy. In addition, it was much lower than that of collagen peptides from tilapia skin (IC_50_ = 2.51 mg/ml) (Sheng et al., 2018), and similar to that of polypeptides from yellowfin tuna (*Thunnus albacares*) head (IC_50_ = 0.24 mg/ml) (Pu et al., 2018). The IC_50_ values for $O_{2}^{\cdot ‐}$ scavenging ability and Fe^2+^ chelating ability were 5.60 and 2.47 mg/ml, respectively, suggesting a relatively weaker activity. But, as the Fe^2+^ chelating ability was higher than that of positive control GSH, no chelating ability was observed in 3 mg/ml of GSH, which was similar to the results of Hu et al. (2012).

**TABLE 1:** The ABTS.^+^ scavenging capacity, $O_{2}^{\cdot ‐}$ scavenging capacity, and Fe^2+^ chelating ability of grass carp hydrolysates (GCHs) prepared under the optimal conditions

Samples	IC_50_ value, mg/mL		
	ABTS.^+^ scavenging ability	$O_{2}^{\cdot ‐}$ scavenging ability	Fe^2+^ chelating ability
GCHs	0.21 ± 0.00^c^	5.60 ± 0.07^d^	2.47 ± 0.09^b^
F1	2.77 ± 0.02^f^	3.48 ± 0.07^c^	ND
F2	0.23 ± 0.01^c^	11.32 ± 0.31^e^	ND
F3	0.11 ± 0.00^b^	0.47 ± 0.01^b^	ND
F4	ND	ND	1.04 ± 0.03^a^
F5	0.41 ± 0.01^d^	ND	2.58 ± 0.06^b^
GSH	0.02 ± 0.00^a^	0.12 ± 0.01^a^	ND

Note

*Different letters (a, b, c) in the upper right corner of each data indicate significant difference among the data on the same column (*p* < .05), ND indicates that no activity was detected at the tested concentration.

### 3.2 Amino acid composition of hydrolysates

The amino acid content expressed as mg/100 g GCHs is shown in Table 2. The content of hydrophobic, acidic, basic, and aromatic amino acids was 21.60, 16.66, 11.07, and 7.03 g/100 g sample, respectively. The hydrophobic amino acid accounted for 28.05% of total amino acids, among which leucine (Leu), alanine (Ala), and valine (Val) were the major components. The antioxidant activity of peptides was reported to be largely correlated with a high proportion of hydrophobic amino acids in their sequence (Liu et al., 2016; Zou et al., 2015). Mendis et al. (2005) speculated that hydrophobic amino acids such as proline (Pro), Ala, and Val played an important role in improving the antioxidant activity of peptides from jumbo squid skin gelatin. Guo et al. (2009) indicated that peptides with Val, Leu, isoleucine (Ile), and Ala at their N‐terminal showed strong antioxidant ability. Furthermore, GCHs were rich in acidic amino acids, in which glutamic acid (Glu) gave the highest content, followed by aspartic acid (Asp). The results were similar to those of a previous finding on grass carp protein hydrolysates (Zhang et al., 2018). Asp and Glu contributed to the antioxidant capacity of peptides because of their negatively charged side chain groups, which can quench unpaired electrons and radicals by providing protons (He et al., 2013). The GCHs also possessed a relatively high content of lysine (Lys) and arginine (Arg), with the values of 5.68 and 3.54 g/100 g sample. Arg and Lys also played an important role in the antioxidant ability of peptides. Tkaczewska et al. (2020) found that Arg and Lys might be the predominant contributors to the radical scavenging properties of *Cyprinus carpio* skin gelatin peptide fraction.

**TABLE 2:** The amino acid composition of grass carp meat hydrolysate (g/100 g sample)

Amino acid	Content	Amino acid	Content
Glu^c^	9.87 ± 0.16	Leu^a^	5.82 ± 0.08
Asp^c^	6.79 ± 0.16	Ala^a^	3.53 ± 0.04
Trp^b^	2.87 ± 0.05	Ile^a^	2.85 ± 0.02
Tyr^b^	1.53 ± 0.05	Met^a^	1.52 ± 0.07
Phe^a,b^	2.63 ± 0.03	Pro^a^	1.93 ± 0.07
Lys^d^	5.68 ± 0.03	Gly^a^	2.84 ± 0.03
His^d^	1.85 ± 0.01	Ser	2.56 ± 0.05
Arg^d^	3.54 ± 0.01	Cys	0.27 ± 0.01
Val^a^	3.32 ± 0.04	Total	59.40 ± 0.71

Note

a, b, c, and d indicate that this amino acid belongs to hydrophobic, aromatic, acidic, and basic amino acids, respectively.

### 3.3 Molecular weight distribution of hydrolysis

**FIGURE 2:**
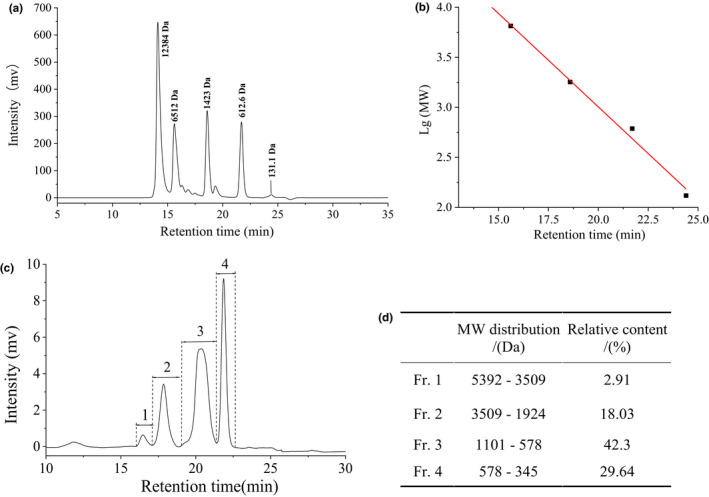
The size exclusion chromatography (a) and molecular weight distribution fitting curve of standards (b), and size exclusion chromatogram (c) and molecular weight distribution (d) of grass carp hydrolysates (GCHs)

The size exclusion chromatogram and molecular weight (MW) distribution curve (B) of standards are shown in Figure 2a,b, respectively. The retention time and log (lg) MW were applied to obtain calibration curve equation: lg (MW) = 6.73474–0.18652 t, *R* ^2^ = .99219. High *R* ^2^ value suggested the reliability of the equation. The size exclusion chromatogram of GCHs and its MW distribution calculated by the calibration curve equation are shown in Figure 2c,d.

It was clear that four fractions were separated from GCHs with the MW ranged from 5.4 kDa to 0.35 kDa. Based on the peak area, 97.08% of peptides showed a MW less than 3.6 kDa. In addition, fraction 3 with MW of 1.10–0.58 kDa was the main constituent in GCHs, which accounted for 42.3% of the total peptide content, followed by the fraction 4 with MW of 0.58–0.35 kDa (29.6%). These results indicated a high proteolysis efficacy of protamex–alcalase two‐step hydrolysis on grass carp muscle. According to previous researches, higher proportion of smaller peptides with molecular weight less than 1.0 kDa was favorable to antioxidant activity. Lin et al. (2013) found that the chicken protein peptide fraction with MW less than 1 kDa had stronger ABTS.^+^ and ^·^OH scavenging ability when compared to others. He et al. (2013) separated rapeseed protein hydrolysates into four fractions and found that the fractions with MW less than 1 kDa had the strongest $O_{2}^{\cdot ‐}$ and DPPH^·^ scavenging ability.

### 3.4 Antioxidant ability of GCHs fractions

**FIGURE 3:**
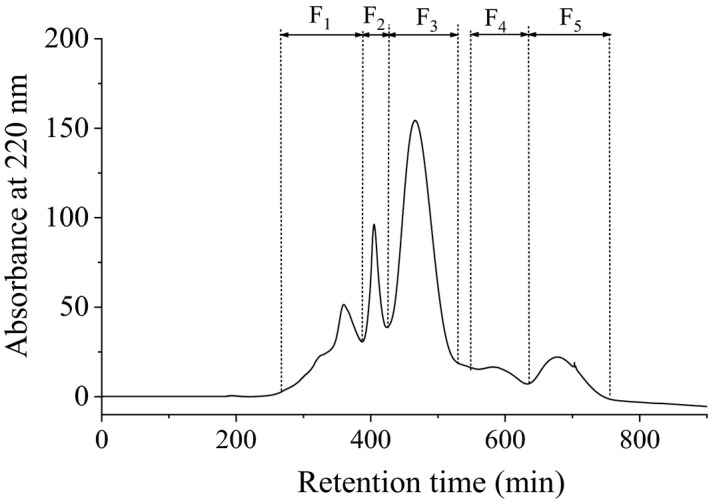
The chromatogram of grass carp hydrolysates (GCHs) separated by Sephadex G‐25

The GCHs were fractionated using Sephadex G‐25 gel filtration column to enrich the peptides with high antioxidant ability. Totally, five fractions were collected, lyophilized, and labeled as F1, F2, F3, F4, and F5 orderly (Figure 3a **)**. Then, all fractions were redissolved in distilled water and used to evaluate the ABTS.^+^ scavenging ability, $O_{2}^{\cdot ‐}$ scavenging ability, and Fe^2+^ chelating ability, and the results are listed in Table 1.

The F3 exhibited the strongest ABTS.^+^ and $O_{2}^{\cdot ‐}$ scavenging ability with the IC_50_ value of 0.11 mg/ml and 0.47 mg/ml, respectively, which was nearly 2‐ and 12‐fold of that of GCHs. The results suggested an excellent enriching effect of the Sephadex G‐25 gel filtration column. The highest radical scavenging ability of F3 could be attributed to its smaller MW distribution. It is well known that longer retention time in Sephadex G‐25 gel filtration column indicates small MW. Zhang et al. (2020) isolated antioxidant peptides from snakehead soup digestion products and found that peptide fraction possesses smaller MW (<2 kDa), which showed significantly stronger antioxidant activity than that with higher MW. Ren et al. (2008) also found that peptides with MW less than 3 kDa contribute more to the antioxidant activity than polypeptides. But the scavenging efficacy of F3 was also higher than those of F4 and F5, which may have resulted from the occurrence of small peptides or free amino acids with low or without antioxidant abilities. The radical scavenging ability of the fractions with MW <3 kD from duck breast hydrolysates showed a negative correlation with its molecular weight (Li et al., 2020). F4 exhibited the best Fe^2+^ chelating ability, but no ABTS.^+^ and $O_{2}^{\cdot ‐}$ scavenging ability was detected when the concentration was set at 10 mg/ml. Therefore, F3 was selected for further peptide identification and antioxidant peptide screening.

### 3.5 Identification and screening of antioxidant peptides in F3

**FIGURE 4:**
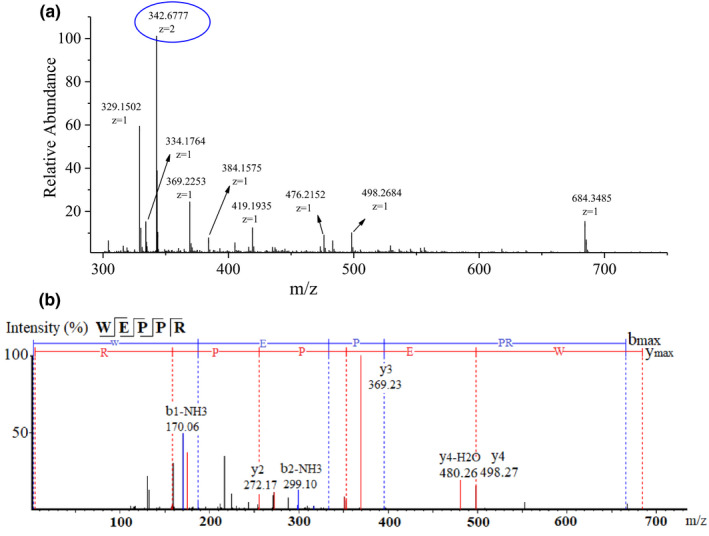
Identification of peptides in F3 by de novo sequence. (a) Full mass spectrometry (MS) scan spectrum at 46.58 min. (b) MS/MS spectrum of ion with m/z at 342.6777^2+^

The amino acid sequence and MW of peptides in F3 were analyzed by Nano‐LC‐Orbitrap‐MS/MS. The MS and MS/MS data were elucidated by de novo sequencing, which was performed based on the b‐series and y‐series ions generated by HCD cleavage. The full MS scan spectrum at 46.58 min and MS/MS spectrum of the ion at m/z 342.6777^2+^ are shown in Figure 4. It was determined to be Trp‐Glu‐Pro‐Pro‐Arg (WEPPR) by matching the data with those recorded in database. Based on the same identification methods, 26 peptides with ALC >95% were identified from F3, and the detailed information is listed in Table 3. The MW distribution of all identified peptides ranged from 408.2099 to 880.3926 Da, and none of them have been previously recorded in the BIOPEP Bioactive Peptide Database (http://www.uwm.edu.pl/biochemia/index. php/en/biopep).

**TABLE 3:** The peptides identified from F3 by LC‐Q‐Orbitrap‐MS/MS

No	Amino acid sequence	RT (min)	ALC (%)	m/z	Mass	Local confidence (%)
1	WEPPR	46.58	99	342.6776	683.3391	99 100 100 100 100
2	APPAMW	54.60	99	336.6611	671.3101	100 100 100 100 99 100
3	WGLDK	53.5	99	309.6668	617.3173	100 100 100 100 99
4	WDAPK	37.47	99	308.6584	615.3016	99 99 100 100 99
5	WDAPR	39.65	99	322.6622	643.3078	99 99 100 100 99
6	FDDLPR	56.37	99	381.6935	761.3708	99 100 100 99 99 99
7	WVPPR	48.44	99	327.6910	653.3649	99 99 100 99 97
8	STHPW	39.76	98	314.1483	626.2812	99 98 99 100 100
9	YPLEAH	36.76	98	365.1827	728.3493	98 98 100 100 99 98
10	LLPDDGDH	38.30	98	441.2048	880.3926	99 98 100 99 99 98 99 98
11	WEAPR	40.88	98	329.6703	657.3234	98 99 99 99 98
12	NPSRPW	45.62	98	378.6939	755.3715	96 99 99 99 99 99
13	FYYGK	42.80	98	339.1692	676.3220	97 98 99 100 100
14	WRPPL	84.6	98	334.6984	667.3806	99 99 100 98 97
15	LLLYK	69.87	98	325.2187	648.4210	90 100 100 100 100
16	LGGY	36.88	97	409.2094	408.2009	96 96 100 100
17	WEPPK	44.34	97	328.6748	655.3329	98 99 100 100 90
18	LFGY	83.17	97	499.2567	498.2478	98 95 99 97
19	NGPWEK	37.59	97	365.6807	729.3445	89 95 100 99 100 100
20	DFRPW	86.01	96	360.6777	719.3391	98 97 97 95 97
21	WETPR	41.52	96	344.6755	687.3340	94 97 98 97 97
22	VAGW	57.35	96	432.2259	431.2169	95 90 99 100
23	LEAPPLH	71.05	96	388.7200	775.4228	96 99 100 100 98 95 85
24	WPEPR	49.99	95	342.6779	683.3391	98 94 98 96 93
25	VEYH	59.12	95	547.2534	546.2438	93 95 99 96
26	DWQPR	37.72	95	351.1722	700.3293	90 90 95 99 100

In this research, the potential antioxidant peptides were selected and synthesized based on the following well‐known structure–activity relationships: (1) Peptides contain hydrophobic amino acid residues, such as Ala, Pro, Leu, Ile, and phenylalanine (Phe), which can increase the accessibility of peptides in water–lipid interface and promote the quenching on free radicals (Cai et al., 2015; Zou et al., 2015). (2) The presence of aromatic amino acids of tyrosine (Tyr) and tryptophan (Trp), which can act as good hydrogen donors and exhibit strong radical scavenging activity (Liu et al., 2016). (3) The presence of basic or acidic amino acids of Arg, Lys, histidine (His), Asp, and Glu, which are able to chelate metal ions through the carbonyl and amino groups in the side chain (Saiga et al., 2003). In addition, the imidazole ring in the R group of His has the ability of donating hydrogen, trapping lipid peroxyl radical, and chelating metal ion (Liu et al., 2016). (4) The presence of cysteine (Cys), the sulfhydryl (SH) group in the R group can act as radical scavenging (Li et al., 2020). Finally, a total of 15 potential antioxidant peptides were screened for further synthesis and bioactivity evaluation, and the physical and chemical properties of these synthesized peptides are shown in Table 4.

**TABLE 4:** Physiochemical properties of the 15 synthesized peptides identified in grass carp hydrolysates (GCHs)

No	Amino acid sequence	MW (Da)	Purity (%)	pI^b^	Net charge^b^	AH^a^ (GRAVY)
P_1_	WEPPR	683.3391	95.76	6.0	0	−2.42
P_2_	WVPPR	653.3649	98.84	9.75	+1	−0.88
P_3_	WEAPR	657.3234	99.80	6.0	0	−1.74
P_4_	WEPPK	655.3329	98.29	6.0	0	−2.30
P_5_	WETPR	687.3340	96.24	6.0	0	−2.24
P_6_	VEYH	547.2534	98.45	5.10	−1	−0.95
P_7_	VAGW	431.2169	99.05	3.57	0	1.18
P_8_	LEAPPLH	775.4228	98.31	5.24	−1	−0.07
P_9_	LFGY	498.2478	99.15	3.61	0	1.23
P_10_	FYYGK	676.3220	99.11	8.50	+1	−0.82
P_11_	APPAMW	671.3101	95.42	5.57	0	0.23
P_12_	LGGY	408.2009	99.72	3.61	0	0.43
P_13_	LLLYK	648.4210	96.44	8.59	+1	1.24
P_14_	LLPDDGDH	880.3926	98.21	3.93	−3	−1.01
P_15_	FDDLPR	761.3708	99.42	4.21	−1	−1.08

Abbreviations: AH, Averaged hydrophilicity; pI, isoelectric point.

^a^ The proportion of hydrophobic amino acids calculated based on the proportion of alanine (Ala), proline (Pro), isoleucine (Ile), leucine (Leu), methionine (Met), phenylalanine (Phe), valine (Val), and tryptophan (Trp) in the peptide.

^b^ The pI and net charge of peptides with amino acids equal to or greater than 5 were calculated with https://web.expasy.org/protparam/, while those of peptides with amino acids less than 5 were calculated with https://pepcalc.com/ppc.php.

### 3.6 **ABTS·^+^** scavenging ability of synthetic peptides

**FIGURE 5:**
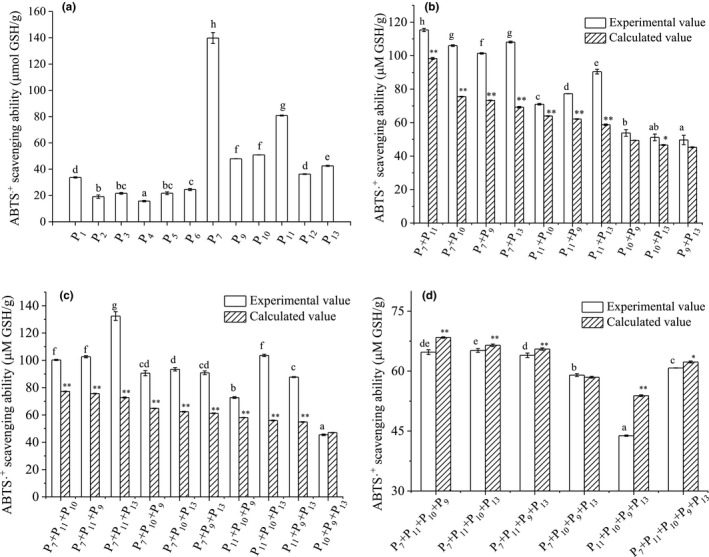
The ABTS.^+^scavenging ability of synthetic peptides (a), and the synergistic and antagonistic effects among selected peptides (b–d). Different letters (a, b, c, etc.) in Figure 4a indicate significant difference among peptides (*p* < .05). Different letters (a, b, c, etc.) in Figure 4b‐d indicate significant difference among the experimental values of varied peptide combinations (*p* < .05). ** and * indicate extremely significant (*p* < .01) and significant (*p* < .05) differences between the experimental and calculated value of the same combination

To compare the antioxidant ability of synthesized peptides intuitively, the ABTS.^+^ scavenging ability of peptides was expressed as μmol GSH equivalent per gram of peptide (μmol GSH/mg), while a higher value suggests stronger antioxidant ability. As shown in Figure 5a, 12 peptides had considerable ABTS.^+^ scavenging ability, all of them containing Trp and Tyr residues. The indolic and phenolic groups in Trp and Tyr can serve as hydrogen donors, contributing to the ABTS.^+^ scavenging ability of peptides. Ledesma et al. (2007) evaluated the ABTS.^+^ scavenging ability of 11 peptides identified from human milk, while the significant radical scavenging capacity was found only in one peptide containing Trp (WSVPQPK) and four peptides containing Tyr (QVVPYPQ, HQIYPV, PYPQ, IYPF). Cai et al. (2015) isolated three antioxidant peptides (PYSFK, GFGPEL, VGGRP) from grass carp skin protein hydrolysates, peptide PYSFK was found to possess the strongest DPPH· and ABTS.^+^ scavenging ability, which may be owed to the presence of a Tyr amino acid residue. However, no ABTS.^+^ scavenging ability was detected in P8, P14, and P15, which may result from the absence of antioxidant amino acid residues such as Trp, Tyr, Cys, and Met in their sequence. Zheng et al. (2016) systematically synthesized 32 dipeptides to study their structure–activity relationships. They found that dipeptides with Trp and Tyr showed the strongest free radical scavenging activity, followed by the dipeptides containing Cys and Met residues.

However, the ABTS.^+^ scavenging ability of the 12 peptides with Trp and Tyr was different, indicating the importance of amino acid sequence. It was apparent that the peptides containing Trp or Tyr residue at the C‐terminus had higher scavenging activity. For example, P7 (VAGW) showed the highest scavenging ability with the value of 139.77 μmol GSH/g, which was followed by P11 (APPAMW) (80.83 μmol GSH/g).. This was consistent with the results found by Saito et al. (2003). Meanwhile, there is no significant difference between P10 and P9 (*p* > .05), which may be due to the presence of two Tyr residues in P10 (FYYGK), enhancing its ABTS.^+^ scavenging ability to a certain degree. The equivalent value of P12 (LGGY) was significantly lower than that of P9 (LFGY), indicating that Phe attached to the N‐terminal Leu contributed more to the ABTS.^+^ scavenging ability of L‐X‐GY than Gly. Among the five similar pentapeptides P1–P5, P1 (WEPPR) and P4 (WEPPK) had the highest and lowest equivalent value, respectively, implying that Arg contributes more to the ABTS.^+^ scavenging ability than Lys in the peptide sequence WEPP‐X.

### 3.7 Synergetic effect of synthetic peptides

Usually, the isolation and purification of protein hydrolysates with antioxidant activity may result in three different results: (1) A minimum of one separated fraction or purified peptide has stronger antioxidant activity than the crude hydrolysates. (2) A minimum of one fraction showed better bioactivity than the hydrolysates, but the purified peptides exhibited weaker activity. (3) The separated fractions gave lower antioxidant ability than the hydrolysate (Zou et al., 2015). For example, the ABTS.^+^ and ·OH scavenging ability, and suppressing lipid oxidation of peach protein hydrolysates (MW >5 kDa, 3–5 kDa, and <3 kDa) were all reduced after ultrafiltration (Vásquezvillanueva et al., 2016). But the antioxidant ability of fraction and peptides from duck breast protein hydrolysates was significantly enhanced after fractionation and purification (Li et al., 2020).

In this research, the radical scavenging ability of F3 was much higher than those of GCHs, but the purified peptides presented much weaker ability, suggesting the presence of synergistic effect among peptides. The combination of two, three, four, and five peptides among P_7_ (VAGW), P_11_ (APPAMW), P_9_ (LFGY), P_10_ (FYYGK), and P_13_ (LLLYK) (the top five activity) was designed to investigate the synergistic effect. The ABTS.^+^ scavenging ability of the combinations with two and three peptides is shown in Figure 5b‐c. Excepting for the combination of P_9_ + P_10_ + P_13_, no antagonism was observed. All the combinations with P_7_ and/or P_11_ exhibited significant synergistic effect (*p* < .05), indicating that P_7_ and P_11_ synergized greatly with other tested peptides. This could result from the role of C‐terminal Trp (W) (Zou et al., 2015). Among the two peptides’ combination, the P_7_ + P_11_ presented the highest ABTS.^+^ scavenging ability, with the GSH equivalent of 115.36 μmol GSH/g. The strongest synergism was found in P_7_ + P_13_, the EV was 38.93 μmol GSH/g higher than the CV. For three peptides’ combination, P_7_ + P_11_ + P_13_ exhibited the strongest ABTS.^+^ scavenging ability and synergism, the EV was 132.42 μmol GSH/g, which was 59.66 μmol GSH/g higher than the CV. In addition, the combination of P_7_ or P_7_ + P_11_ with P_13_ always showed significantly higher synergism than the combination with P_9_ and P_10_ (*p* < .05). The above results indicated strong synergistic effect among P_7_, P_11_, and P_13_ again, which could be due to the fact that P_13_ possesses 3 Leu and 1 Lys, while P_9_ and P_10_ contain only 1 Lys and 1 Leu, respectively. Zhang et al. (2017) investigated the synergistic effect between amino acids by the ABTS.^+^ scavenging ability model and found that Trp synergized significantly with Leu, Lys, and Arg (*p* < .05).

Unexpectedly, except for P_7_ + P_10_ + P_9_ + P_13_, no synergistic effect was observed on the 4 or 5 peptides’ combination among P_7_, P_9_, P_10_, P_11_, and P_13_ (Figure 5d). Controversially, except for P_7_ + P_10_ + P_9_ + P_13_, all tested four or five peptides’ combination exhibited different degrees of antagonism. The EV was significantly lower than the corresponding CV (*p* > .05). Among which, P_10_ + P_9_ antagonized P_11_ + P_13_ most, the EV was 10 μmol GSH/g lower than the CV. In addition, the ABTS.^+^ scavenging ability of P_7_ + P_11_ + P_13_ was greatly decreased when P_10_ or P_9_ was included, the GSH equivalent value was reduced by 51%~52%, suggesting that the presence of P9 or P10 could reduce the radical scavenging ability of P_7_ + P_11_ + P_13_ greatly. This could be due to the fact that the presence of P9/P10 suppressed the proton‐donating ability of P_7_ + P_11_ + P_13_, leading to reduced radical scavenging ability, but the detailed mechanism needs further research.

## 4 CONCLUSIONS

In this study, the two‐step enzymatic hydrolysis of grass carp muscle for preparing antioxidant peptides was optimized. The optimal conditions were: first‐step hydrolysis with protamex at a protein–liquid ratio of 4%, enzyme/substrate ratio of 10,000 U/g, pH of 7.0, and temperature of 50°C for 3 h, followed by the second‐step hydrolysis with alcalase at the following conditions: pH, 9.0; enzyme/substrate ratio, 6,000 U/g; temperature, 50°C for 2 h. The obtained GCHs mainly consisted of hydrophobic and acidic amino acids with MW lower than 5.4 kDa. Twelve novel antioxidant peptides were identified from the most active fraction (F3), among which VAGW possessed the highest ABTS.^+^ scavenging activity (139.77 μmol GSH/g). Significantly synergistic effects were observed between the combination of two and three peptides, VAGW, APPAMW, and LLLYK, which exhibited the strongest synergism with the experimental value of 59.66 μmol GSH/g higher than the calculated. The C‐terminal Trp played an important role in the synergism. In conclusion, fraction 3 of GCHs and VAGW have a promising potential to serve as natural antioxidants used in functional materials for a supplement.

## CONFLICT OF INTEREST

The authors declare no competing financial interests.

## Supporting information

Figure S1

## Data Availability

The data that support the findings of this study are openly available
