# Supplementary material for: Extraction optimization and screening of antioxidant peptides from grass carp meat and synergistic–antagonistic effect
Source: Food Sci Nutr. 2022 Mar 25;10(5):1481–93. doi: 10.1002/fsn3.2765 (PMC9094479; doi:10.1002/fsn3.2765)
Supplement: Supplementary file 1 — Figure S1 [file FSN3-10-1481-s001.docx]

**FIGURE** 1S Effect of double enzyme combination on the ABTS·^+^ scavenging ability of grass carp muscle hydrolysates
